# Regression of retinal capillary hemangioblastoma with systemic belzutifan in von Hippel–Lindau disease: a case report

**DOI:** 10.3389/fonc.2026.1870341

**Published:** 2026-07-09

**Authors:** Angelica Aja Dangca Alcoreza, Cheryn Song, Junyeop Lee

**Affiliations:** 1 Department of Ophthalmology, Quirino Memorial Medical Center, Quezon City, Philippines; 2 Department of Ophthalmology, Asan Medical Center, University of Ulsan College of Medicine, Seoul, Republic of Korea; 3 Department of Urology, Asan Medical Center, University of Ulsan College of Medicine, Seoul, Republic of Korea

**Keywords:** belzutifan, HIF-2α inhibitor, renal cell carcinoma, retinal capillary hemangioblastoma, von Hippel-Lindau disease

## Abstract

**Purpose:**

To report a case of retinal capillary hemangioblastoma (RCH) regression in a patient with von Hippel–Lindau (VHL) disease following treatment with systemic belzutifan.

**Case presentation:**

A 49-year-old female with VHL disease presented with a retinal capillary hemangioblastoma in the left eye that had been previously treated with laser therapy. She subsequently developed a new retinal lesion and interval growth of a renal intraparenchymal mass, for which systemic belzutifan was initiated. Four months after treatment initiation, a reduction in the size of the retinal lesions was observed, along with decreased perfusion and vascularity.

**Conclusion:**

Systemic belzutifan therapy for VHL disease may induce regression of retinal capillary hemangioblastomas and can be effective as either a primary or an adjunctive treatment modality.

## Introduction

Von Hippel–Lindau disease is a hereditary tumor syndrome characterized by a predisposition to multisystem tumors such as hemangioblastoma of the retina and central nervous system, renal cell carcinoma, pheochromocytoma, paragangliomas, and neuroendocrine tumors. Germline mutations of the *VHL* gene result in inactivation of the VHL protein, a tumor suppressor protein, leading to a pseudohypoxic state (1, 2).

Retinal capillary hemangioblastoma is a benign hamartoma that is associated with VHL in up to 85% of patients by age 25 years (3). It usually presents as a round, well-circumscribed, orange lesion on the peripheral retina with dilated and tortuous feeder vessels (3). Known treatment options include observation and ablation with cryotherapy, thermal laser, or photodynamic therapy (2).

Belzutifan is a hypoxia-inducible factor 2 alpha (HIF-2α) inhibitor approved by the Food and Drug Administration for adult patients with VHL-related tumors not requiring urgent surgery (2). It leads to a reduction in cellular proliferation, angiogenesis, and tumor growth and have showed an objective response in 49% of patients with renal cell carcinoma and a 100% improvement in patients with retinal hemangioblastoma in a phase 2 single-group trial (3, 4).

## Case presentation

This is a case of a 49-year-old female with von Hippel–Lindau disease who was referred by the Urology Department for ophthalmic evaluation and monitoring. Past medical history includes renal cell carcinoma, a pancreatic cyst status post aspiration, and multiple cerebellar hemangioblastoma status post resection and Gamma Knife radiosurgery. Twenty years prior, the patient was diagnosed with a retinal capillary hemangioblastoma of the left eye and underwent laser photocoagulation, and was subsequently lost to follow-up. At presentation, she reported intermittent episodes of floaters but was otherwise asymptomatic.

On examination, best-corrected visual acuity (BCVA) was 20/20 in both eyes on the Snellen ETDRS chart. Intraocular pressure was 20 mmHg in the right eye and 21 mmHg in the left eye. Manual refraction revealed −4.25 diopters sphere on the right eye and −5.00 diopters sphere with +1.25 diopters cylinder at axis 125 in the left eye. Anterior segment examination was unremarkable bilaterally.

**Figure 1:**
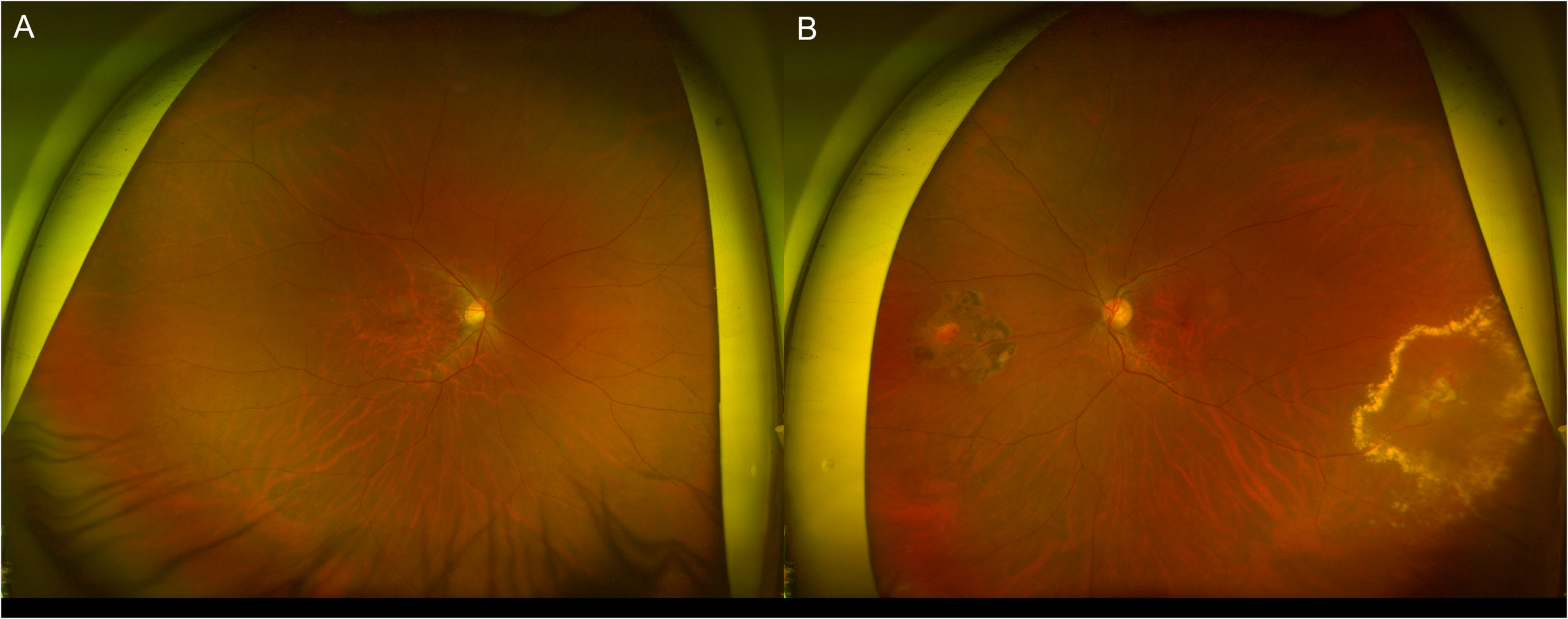
Widefield color fundus photographs of both eyes. The right eye shows no visible retinal capillary hemangioblastoma. The left eye demonstrates multiple retinal capillary hemangioblastoma lesions with associated retinal changes visible on color fundus imaging.

Dilated fundoscopy examination of the right eye demonstrated clear media, a healthy optic disc with distinct disc borders, a cup-to-disc ratio of 0.3, and an arteriovenous ratio of 2:3, with no abnormal lesions identified. Fundus examination of the left eye revealed an orange-yellow nodular lesion 0.138 mm^2^ in area, located on the nasal retina, with prominent feeder vessels and surrounding laser marks consistent with a previously treated retinal capillary hemangioblastoma. Additionally, an incidental irregular yellow lesion measuring 0.113 mm^2^ in area was noted in the inferotemporal peripheral retina, associated with feeder vessels and surrounding exudation and suggestive of a new retinal capillary hemangioblastoma (**Figure 1**).

**Figure 2:**
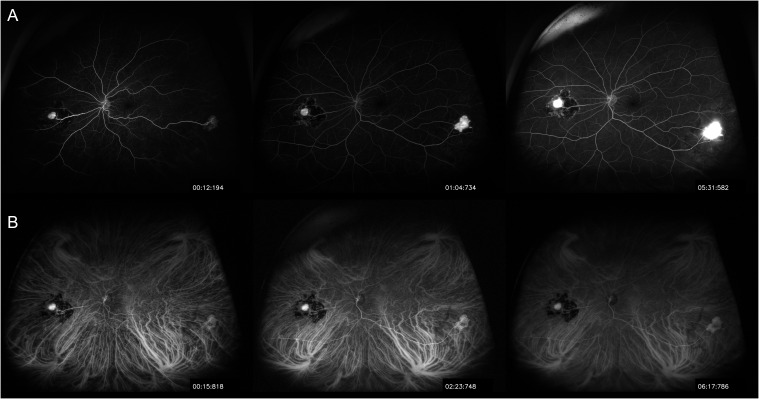
Widefield fluorescein angiography and indocyanine green angiography images of the left eye. Panel A shows early hyperfluorescence of an orange-yellow retinal capillary hemangioblastoma with progressive fluorescein leakage during the late angiographic phases. Panel B shows corresponding indocyanine green angiography images demonstrating persistent hypercyanescent retinal capillary hemangioblastoma lesions throughout all phases of imaging.

Further diagnostic evaluation included optical coherence tomography, which demonstrated hyperreflectivity on the retinal surface of the left eye, consistent with an epiretinal membrane. Fluorescein angiography (FA) revealed early well-demarcated hyperfluorescence of the orange-yellow lesion with progressive leakage in the late phases. Indocyanine green angiography (ICGA) showed a hypercyanescent lesion that remained visible throughout all phases of the study (**Figure 2**).

Cryotherapy was initially planned for treatment of the newly identified lesion; however, the patient was managed with close observation after initiation of belzutifan, an oral hypoxia-inducible factor-2 alpha (HIF-2α) inhibitor prescribed at 120 mg daily by the primary service for a slowly growing right intraparenchymal renal mass. This approach provided an opportunity to evaluate the effect of belzutifan on the retinal capillary hemangioblastoma while reserving conventional local therapies as potential rescue treatment modalities should lesion progression occur.

At the 2-month follow-up after initiation of belzutifan, the patient had elevated liver function test results (ALT 230 IU/L; AST 103 IU/L; alkaline phosphatase 142 IU/L); hence, the belzutifan dosage was reduced to 80 mg daily, with the addition of ursodeoxycholic acid 100 mg four times daily to support liver function. On repeat examination, the patient’s visual acuity was 20/16 in both eyes. The primary and secondary lesions were also noted to decreased in size from 0.138 mm^2^ to 0.082 mm^2^ and from 0.113 mm^2^ to 0.083 mm^2^, respectively. The lesions demonstrated regression with no evidence of progression.

**Figure 3:**
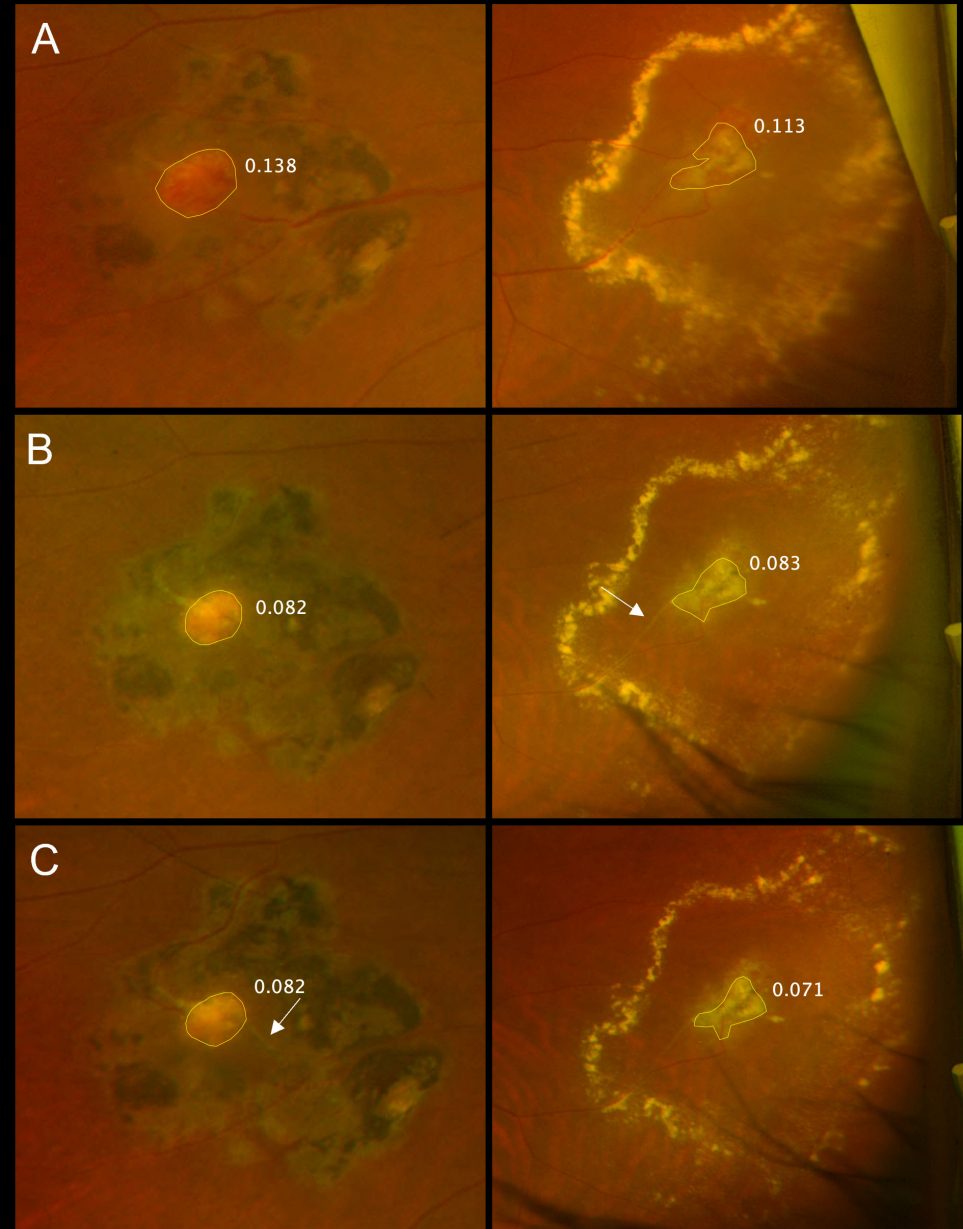
Serial color fundus photographs of the left eye obtained at baseline and 2 and 4 months after initiation of systemic belzutifan therapy. The images focus on retinal capillary hemangioblastomas and demonstrate interval changes following treatment. An arrow highlights a ghost vessel, indicating markedly reduced blood flow within the lesion's feeding vessel after treatment.

**Figure 4:**
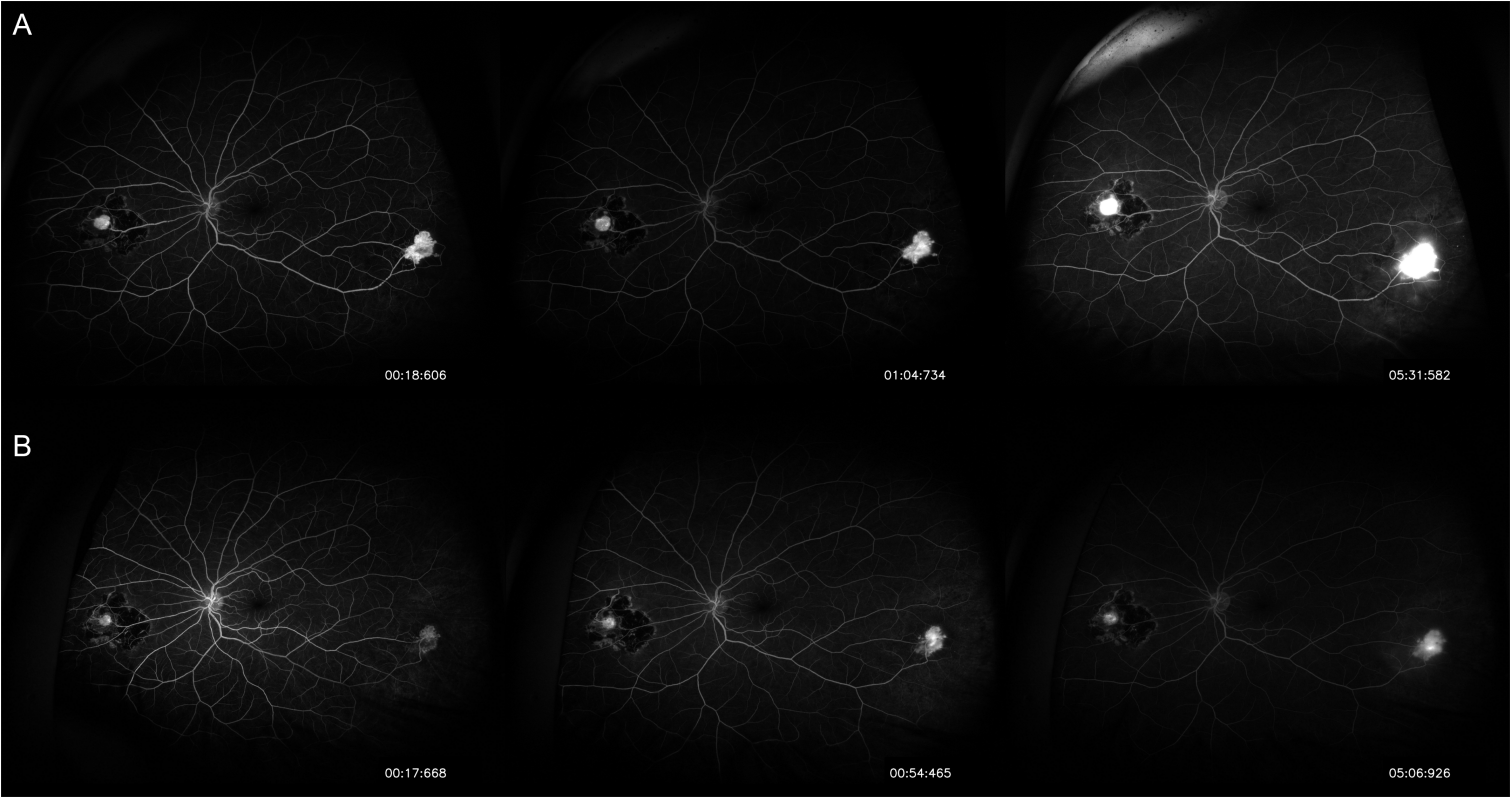
Two panels compare widefield fluorescein angiography images of the left eye obtained at baseline Panel **(A)** and 4 months after initiation of belzutifan treatment Panel **(B)**. Each panel includes sequential angiographic images demonstrating the retinal vasculature and a peripheral retinal capillary hemangioblastoma. Compared with baseline, the follow-up images show decreased intensity of hyperfluorescence in the lesion, consistent with reduced fluorescein leakage and decreased tumor activity.

At four and six months after initiation of belzutifan therapy, a noticeable reduction in perfusion and vascularity of the retinal hemangioblastomas was observed on clinical examination and imaging, with markedly reduced blood flow in the feeder vessels progressing to ghost vessels. Lesion sizes were noted to be 0.082 mm^2^ and 0.071 mm^2^ (**Figures 3**, **4**). The patient’s visual acuity remained 20/16 in both eyes. Given this favorable response, local ablative therapies were deferred to avoid potential treatment-related complications. On repeat magnetic resonance imaging of the kidneys, there was a noticeable decrease in the volume of the cystic lesion in the left kidney, with partial response of the solid lesion on the right kidney. No new metastases were reported.

## Discussion and conclusion

The main risk factors for vision loss associated with retinal capillary hemangioblastoma are tumor location and patient age. Additional contributors include the presence of exudation, vitreous hemorrhage, tractional effects, and neovascular glaucoma (5). The presence of these factors serves as a guide in deciding the treatment modality appropriate for each patient.

Fairbanks et al. described a case of multiple leaking peripheral retinal capillary hemangioblastomas treated with belzutifan, which showed improvement in vascular engorgement and tortuosity as well as a reduction in size after 6 months (6). Similarly, Mustafi et al. presented a pediatric patient with juxtapupillary RCH associated with a macula-involving exudative retinal detachment. The patient received daily belzutifan for 6 months and showed signs of improvement in vascular tortuosity and a decrease in exudative retinal detachment (7). Another case report by Aykut et al. described a 15-year-old patient with RCH associated with an exudative tractional retinal detachment in one eye and a small RCH in the other eye. After one year of treatment, vascular tortuosity was noted in the better-seeing eye, while fibrosis with subretinal fluid resolution was observed in the poorer-seeing eye (8). Based on the observed clinical changes, belzutifan in our patient may not only directly target tumor vessels but also induce changes in the feeding vessels supplying blood to the tumor, thereby exerting a dual mechanism of action that leads to suppression of the hemangioma. Previous reports have described reduced vascularity of retinal hemangioblastomas after belzutifan treatment, including narrowing or diminution of feeding vessels, generally interpreted as secondary to suppressed angiogenic signaling. In contrast, our case showed a more pronounced change, with marked flow reduction and near ghost-vessel transformation of the feeding vessel. These findings suggest that belzutifan may exert a dual effect by directly suppressing tumor vasculature while also inducing significant changes in the feeding vessels. This distinctive vascular response underscores the clinical significance of the present case.

Our patient presented with non-visually threatening peripheral lesions with exudation, warranting initial treatment with laser photocoagulation or cryotherapy. However, with the advent of a novel treatment option, belzutifan, approved for the management of VHL disease-associated renal cell carcinoma, CNS hemangioblastoma, or pancreatic neuroendocrine tumors, its use was deemed favorable in our case.

This phenomenon can be explained in the context of HIF-2α signaling. HIF-2α is a key transcriptional regulator of angiogenic and metabolic pathways that maintain endothelial cell survival, vascular tone, and perfusion, particularly in highly vascularized tumors. Inhibition of HIF-2α by belzutifan suppresses downstream targets such as VEGF, ANGPT2, and other hypoxia-responsive genes, leading not only to regression of the tumor microvasculature but also to functional destabilization of feeding vessels that are critically dependent on sustained HIF-2α signaling (9). Consequently, reduced endothelial viability and impaired vascular maintenance may result in profound flow attenuation and ghost-vessel-like transformation, as observed in our case.

Ercanbrack et al. presented a case series of patients treated with belzutifan in conjunction with photocoagulation therapy. Among three patients described, all had a significant reduction in both the size and vascularity of the lesions (2). Similar to the previously reported cases, our patient demonstrated regression of the lesions at the four-month follow-up after initiation of therapy. It is also noteworthy that, when comparing the primary lesion treated with photocoagulation prior to belzutifan initiation and the treatment-naive secondary lesion, both showed a significant amount of regression in terms of perfusion and vascularity, highlighting the efficacy of belzutifan therapy as an adjunctive and a primary treatment.

Although belzutifan has demonstrated promising efficacy in VHL-associated retinal capillary hemangioblastomas, the optimal treatment duration remains undefined. Current practice is largely guided by clinical response, tolerability, and multidisciplinary assessment. Clinicians should be aware of the drug’s known adverse effect profile, particularly anemia, fatigue, and laboratory abnormalities, which warrant periodic systemic monitoring. In our patient, treatment was complicated by elevated liver function test results, warranting a reduction in the belzutifan dose from 120 mg to 80 mg daily while maintaining therapeutic response. Furthermore, recurrence or progression of hemangioblastomas after treatment discontinuation has been reported, suggesting that sustained disease control may require ongoing surveillance and, in selected cases, prolonged therapy. As our patient remains on treatment with a follow-up duration of six months, the long-term durability of response after drug cessation cannot be assessed. Additional studies are needed to establish evidence-based recommendations regarding treatment duration, discontinuation criteria, retreatment strategies, and long-term follow-up protocols.

This case highlights the potential role of belzutifan therapy as either a primary or an adjunctive treatment option for retinal capillary hemangioblastoma in selected patients with VHL disease. Close monitoring remains essential, and belzutifan may be particularly valuable for lesions in visually critical locations where conventional treatments such as laser photocoagulation or cryotherapy carry a risk of vision-threatening complications.

## Data Availability

The original contributions presented in the study are included in the article/supplementary material. Further inquiries can be directed to the corresponding author.
